# Regional Variations in Pesticide Residue Detection Rates and Concentrations in Saudi Arabian Crops

**DOI:** 10.3390/toxics11090798

**Published:** 2023-09-21

**Authors:** Majed S. Alokail, Sherif H. Abd-Alrahman, Abdullah M. Alnaami, Syed D. Hussain, Osama E. Amer, Manal E. A. Elhalwagy, Nasser M. Al-Daghri

**Affiliations:** 1 Protein Research Chair, Department of Biochemistry, College of Science, King Saud University, Riyadh 11451, Saudi Arabia; 2 Central Agricultural Pesticide Laboratory, Department of Pesticides Residues and Environmental Pollution, Agricultural Research Center (ARC), Dokki, Giza 12618, Egypt; 3 Chair for Biomarkers of Chronic Diseases, Department of Biochemistry, College of Science, King Saud University, Riyadh 11451, Saudi Arabia; 4 Central Agricultural Pesticide Laboratory, Department of Mammalian Toxicology, Agricultural Research Center (ARC), Dokki, Giza 12618, Egypt

**Keywords:** persistent organic pollutants, cypermethrin, Saudi Arabia, agriculture products

## Abstract

There is a scarcity of evidence on the levels of pesticide residues among common crops grown in the different regions of the Kingdom of Saudi Arabia (KSA). The present study aims to fill this gap. We collected samples across four regions of KSA (N = 41 from the west, N = 146 from the central, N = 131 from the north and N = 74 samples from the east). Food samples were extracted and cleaned using the modified quick, easy, cheap, effective, rugged and safe (QuEChERS) methodology. Tandem mass (LC-MS/MS and GC-MS/MS) was used to detect pesticide residues. The highest pesticide residue detection rate was 89.7% in the central region, followed by 88.5% in the north, 83.8% in the east and 70.7% in the western region (*p* = 0.01). Pesticide residue detection rates were significantly higher in fruits than vegetables (*p* = 0.02). Cypermethrin detection was most common overall, particularly in the Western region (*p* = 0.002), and pyraclostrobin concentration was the highest among all residues investigated. In conclusion, high detection rates of moderately hazardous pesticide residues were found in various crops across regions in KSA. Routine biomonitoring programs across KSA regions should be implemented, as well as public health campaigns to decrease pesticide residue consumption and exposure.

## 1. Introduction

Persistent organic pollutants (POPs) and pesticide residues are widely known toxic chemicals because of their ability to detrimentally accumulate both in the human body and the environment [1]. They are considered highly lipophilic, hence the potential to accrue in most living organisms, especially within the food chain, including plants, fishes, birds and mammals [2]. Hence, the POPs’ resistance to biodegradation has made them a clear concern for food safety. In fact, exposure to POPs, even at a minimum, has been implicated in various health issues, most notably in terms of endocrine disruption, birth defects, dysfunctional immuno-metabolism and chronic conditions such as malignancies, cardiovascular and endocrine disease, to name a few [3]. It was only in 1995 that global measures spearheaded by the United Nations Environment Programme (UNEP) substantially regulated and prohibited the use of POPs, and these have since been enforced and adopted by the Stockholm Convention, which, as of 2022, has 185 participating states and the European Union [4]. 

Pesticide residues, on the other hand, where some POPs are classified, are also considered biohazard compounds that contribute to environmental stress and deleterious human effects when exposed in various routes such as inhalation, ingestion or skin contact [5]. As of 2020, unintentional acute pesticide poisoning had 385 million cases globally, 11,000 of which were considered fatal [6]. Individuals working in the agricultural sector, including operators and workers, are particularly vulnerable to pesticide exposure, which is predominantly driven by direct application and reentry [7]. General health effects from pesticide toxicity include acute neurologic and respiratory symptoms, as well as more chronic disorders such as malignancies, sterility and impaired immune function [5].

The Kingdom of Saudi Arabia (KSA) was one of the first signatories of the Stockholm Convention in 2002, but, unfortunately, because of the lack of data related to POPs, it has not fully ratified the agreement. Of the various POP compounds listed in the Stockholm Convention, KSA so far ratified only 10 (alpha hexachlorocyclohexane, beta hexachlorocyclohexane, chlordecone, hexabromobiphenyl, hexabromodiphenyl, lindane, pentachlorobenzene, perfluorooctane, endosulfan and tetra/pentabromodiphenyl ether), and this was in the year 2012. Only one compound was added in 2014, as well as three more compounds in 2016, three in 2018 and another three compounds in 2020 [8]. Furthermore, KSA, as a participating member of the WHO Global Strategy for Food Safety, has its share of responsibilities in ensuring that international standards for food safety are met, and this is implemented under the Saudi Food and Drug Administration.

Among the limited biomonitoring studies, one was conducted by Al-Daghri and colleagues (2019) among 302 adult Saudis and found high concentrations of DDT and its metabolites, which were considerably higher in males than females [4]. In terms of soil samples, Al-Wabel and colleagues performed monitoring of major pesticide residues (organochlorine pesticides, organophosphorus, carabamates, pyrethroids and herbicides) using a simple microwave-assisted extraction (MAE) technique in soil samples collected from 15 regions in Saudi Arabia [9]. In their study, they found out that the most common compounds present in the regions were dimethoate, chloroneb, methomyl, oxamyl and toxaphen, with Jizan region having the highest number of contaminated soil samples, followed by the regions of Wadi Al-Dwaser and Abha [9]. Among crops, pesticide residues were mostly studied in dates, a common agricultural export of KSA [10,11]. One study in the region of Al-Qassim investigated pesticide residues in 160 vegetable samples and found that the maximum residue limit (MRL) values exceeded—mostly in cabbage, squash, green pepper and carrots—were for carbaryl, biphenyl and carbofuran, the most commonly found pesticide residues [12]. Given the scarcity of available data, and in the interest of public health safety, it is important to fill important monitoring gaps in the detection of POPs and pesticide residues present in the agricultural products grown and consumed within KSA. To the best of our knowledge, there is little to no evidence on the comparison of levels of pesticide residues among common Saudi crops grown in different regions, and addressing this provides crucial data on the potential human exposure to the omnipresent pesticide residues in Saudi settings. Hence, the present study aims to determine the levels of pesticide residues in the different fruits and vegetables collected from the major regions of KSA. The present study covers some of the compounds listed under the Joint Food Agriculture Organization/World Health Organization (FAO/WHO) Meeting on Pesticide Residues [13].

## 2. Materials and Methods

### 2.1. Study Design and Sampling Location

**Figure 1:**
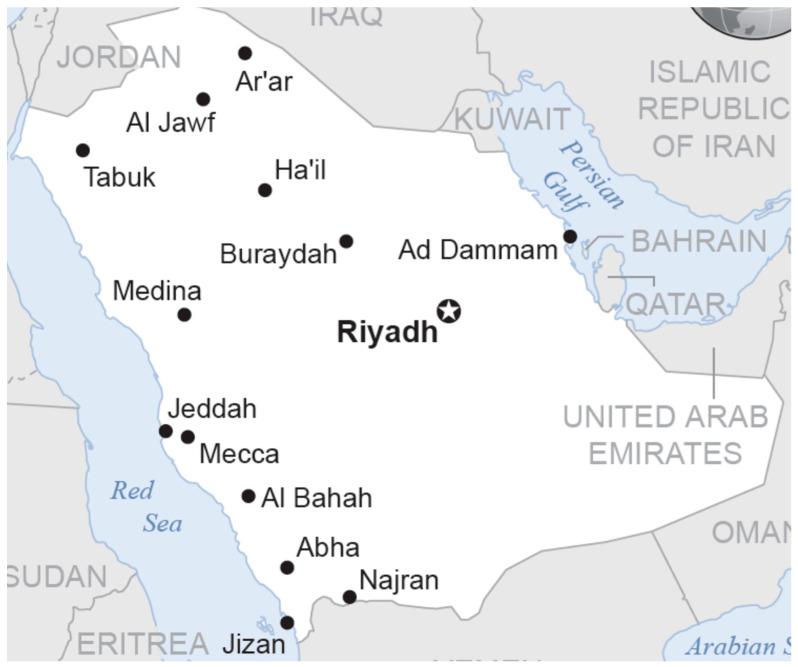
Regions [western (Jeddah), eastern (Dammam), northern (Buraydah) and central (Riyadh)] where produce (fruits and vegetables were purchased).

In the present cross-sectional study, locally produced vegetables and fruits purchased from four different regional provinces were included. The sampling areas represent the major industrial, agricultural and population centers of Saudi Arabia (Figure 1) [14]. The samples were collected according to international monitoring protocols [15,16].

### 2.2. Food (Vegetables and Fruits) Sample Preparation

A total of 28 fruits and vegetables (apple, apricot, arugula, banana, cabbage, cantaloupe, carrot, cucumber, eggplant, fig, grapes, green beans, kiwi, lemon, lettuce, mandarin, orange, peach, pear, pepper, potato, pomegranate, squash, strawberry, tomato and watermelon) were included for sampling. Food samples were extracted and cleaned up using modified QuEChERS methodology (quick, easy, cheap, effective, rugged, and safe) [17,18]. In brief, 1 kg worth of newly purchased vegetable or fruit samples were chopped and homogenized for 5 min at high speed in a laboratory homogenizer. Homogenized sample (15 g) was placed into 50 mL polyethylene tube, then 15 mL of acetonitrile 1% acetic acid was added into each tube. The samples were well shaken using a vortex mixer at maximum speed. Afterwards, 4 g of anhydrous magnesium sulphate and 1.0 g of sodium chloride were added and then extracted by shaking vigorously on vortex for 5 min and centrifuged for 10 min at 5000 rpm. An aliquot of 4 mL was transferred from the supernatant to a new clean 15 mL centrifuge tube containing 100 mg PSA and 500 mg anhydrous magnesium sulphate. The samples were again shaken using a vortex for 1 min and then centrifuged for 5 min at 6000 rpm. An aliquot of 2 mL was concentrated to dryness. All samples were prepared at the Chair for Biomarkers of Chronic Diseases (CBCD) at King Saud University (KSU), Riyadh, Saudi Arabia.

### 2.3. Pesticide Residue Analysis

Mass spectrometer (MS) Agilent Ion Trap Series 240 connected with Agilent 7890 gas chromatograph was used for the determination of 61 unique pesticide residues, hazard classifications of which are presented as Table S1. Results were confirmed with liquid chromatography (LC) with SCIEX 7500-Triple Quadrupole LC-MS System (LC-MS/MS). For each sample, 2 µL was injected to split-splitless injector and analytical capillary column was DB-5MS for separation. A series of selected analytical standards (1.0, 5, 10, 25, 50 and 100 ng mL^−1^) were prepared in n-hexane. Calibration curves were generated by plotting peak area versus concentration. Excellent linearity, good separation and repeatability were observed. Acceptable limit of detection (LOD) was obtained at 1.0–5.0 ng mL^−1^. Samples in duplicates at five levels were spread equally within the analytical range. The calibration curve and recovery validation studies were repeated three times. Rate of recovery for all ranged from 83.5 to 96.4%.

### 2.4. QA/QC Regime

Samples were prepared and analyzed in duplicates for quality control and certified reference standards were used for this purpose. The analytical method performance parameters, which include linearity, repeatability, reproducibility, accuracy, precession, LOD and LOQ, were determined for each tested compound. Series concentration levels were used for the calibration curves. Standard quality assurance protocols were used throughout the study. For external quality control/quality assurance assessment, random sample batches were sent to the Central Agricultural Pesticides Laboratory, Agricultural Research Center (ARC), Giza, Egypt.

### 2.5. Data Analysis

The data was analyzed using SPSS version 21.0 (Chicago, IL, USA). Pesticide residue concentrations were expressed as mean and standard error (SE). Detection rates were presented as frequencies and percentages (%). Mann–Whitney U test and Kruskal–Wallis tests were used to determine differences in concentrations according to type of foods and regions, respectively. Chi-square tests of independence were used to determine significant differences in the detection rates according to food type and regions. A *p* < 0.05 was considered significant.

## 3. Results

All 392 samples were analyzed for the presence of pesticide residues. Overall, only 54 samples (13.8%) were residue-free. Cypermethrin was the most frequent pesticide compound found in the samples, with a prevalence of 8.2%, followed by pyrimethanil (5.6%), indoxacarb (5.1%), imazalil (4.6%), thiamethoxam (4.6%), acetamiprid (4.3%), fludioxonil (4.3%), imidacloprid (3.8%), chlorpyrifos (3.1%) and thiabendazole (3.1%). Other residues detected per sample are listed in Supplementary Table S2.

### 3.1. Regional Distribution of Pesticide Residues

Samples were taken across four regions of KSA (N = 41 from the western region, N = 146 from the central region, N = 131 from the northern region and N = 74 samples from the eastern region) (Table 1). The highest detection rate of pesticide residues was 89.7% in the central region, followed by 88.5% in the northern region, 83.8% in the eastern region and 70.7% in the western region. There was a statistically significant difference between the detection rates of the central and western regions, as well as the northern and western regions, suggesting that both central and northern regions had significantly higher pesticide residue detection rates than the western region. Pesticide residue distribution across the four regions was also stratified according to fruits and vegetables, and no significant differences were observed across regions. 

**Figure 2:**
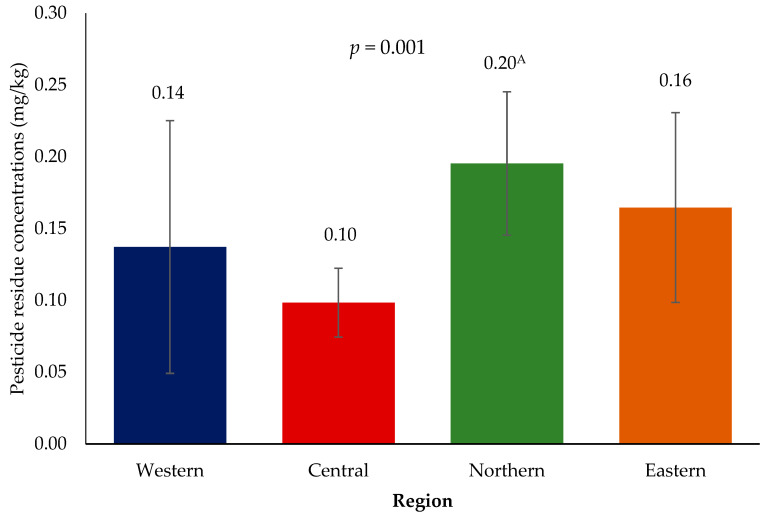
Pesticide residue concentrations (mg/kg) according to regions. Superscript ^A^ denotes significance compared to Central region.

Aggregate concentrations of 33 (54%) out of the 61 pesticide residues detected in at least two regions are listed in Table 2. Of these 33 compounds, only 1 (abamectin) was classified as highly hazardous (class 1b), 20 (61%) were classified as moderately hazardous (class II), 7 (21%) were slightly hazardous (class III) and 5 (15%) were unlikely to present acute hazard (class U). Figure 2 showed that the combined concentration of residues was lowest in the central region with 0.10 ± 0.02 mg/kg, followed by 0.14 ± 0.09 in the western region, 0.16 ± 0.07 in the eastern region and 0.20 ± 0.05 in the northern region (*p* = 0.001). Pyraclostrobin concentrations were recorded in only three samples, one in each region except the western region, but its sum concentration was highest in all the pesticide residues examined (1.76 mg/kg ± 1.34). The highest mean concentration of residues per region were indoxacarb in the western region, fipronil in the central region, pyraclostrobin in the northern region and pyrimethanil in the southern region. The Bonferroni adjusted post hoc analysis showed that the northern region had significantly higher concentration as compared to the western region. Unadjusted analysis also showed that the central region had significantly lower pesticide residue concentration than the northern region; however, this significance disappeared after the Bonferroni adjustment. No differences in residue concentrations were observed across the regions, as reported in Table 2. Descriptive statistics for pesticide residues that were only detected in only one region are presented in Table S3.

### 3.2. Food Type

**Figure 3:**
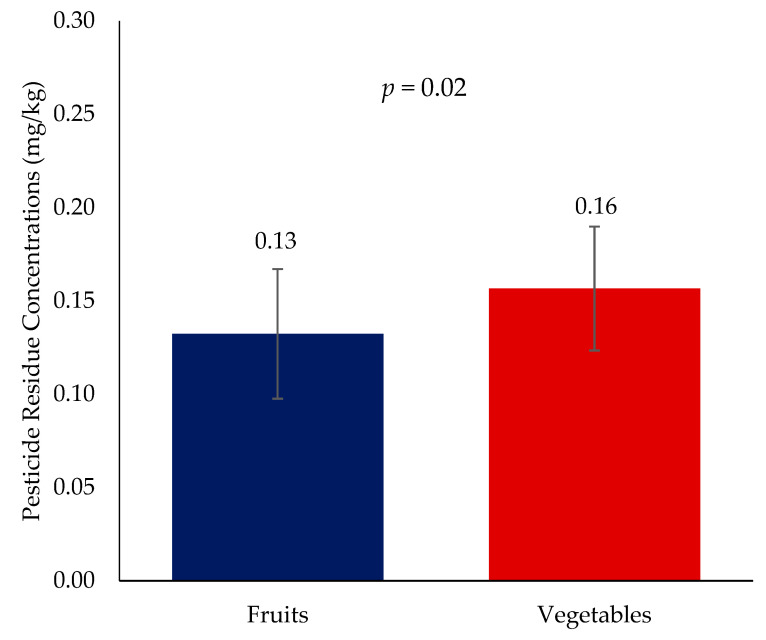
Pesticide residue concentrations (mg/kg) according to food type.

A total of 392 samples obtained across four regions consisted of both fruits (N = 149) and vegetables (N = 243). The detection rate of pesticide residues among fruits and vegetables were 91.3% and 83.1%, respectively (Table S2). Detection rates were significantly higher in fruits than vegetables (0.13 ± 0.04 and 0.16 ± 0.03; *p* = 0.02). Furthermore, in fruits, residue concentrations were highest for indoxacarb (one sample, 0.58 mg/kg), pyrimethanil (0.51 mg/kg ± 0.3), fludioxonil (0.36 mg/kg ± 0.2), fluopyram (0.31 mg/kg ± 0.04) and imidacloprid (one sample 0.24 mg/kg). In vegetables, the highest concentrations detected include pyraclostrobin (2.6 mg/kg ± 1.8), acetamiprid (0.58 mg/kg ± 0.2), indoxacarb (0.52 mg/kg ± 0.2), chlorpyrifos (0.32 mg/kg ± 0.3) and cypermethrin (0.22 mg/kg ± 0.1). Aggregate concentrations and pesticide residues across food types are described in Table 3 and Figure 3. Furthermore, none of the pesticide residues were significantly different across food type (Table 3). The descriptive statistics for residues that were only detected in one food type are presented in Table S4.

## 4. Discussion

The present study investigated the regional variations in the concentrations of pesticide residues in fruits and vegetables purchased in four major regions in KSA and showed that, although fruits and vegetables coming from the western region had about 70% detection rate in pesticide compounds, it is significantly the lowest when compared to its counterpart crops in the central and northern regions. Both of these regions, as well as the eastern region, have more than an 80% detection rate of pesticide residues. The northern region also had the highest aggregate concentration of residues as compared to all other regions investigated. Among the compounds examined, levels of cypermethrin were the most abundant. Lastly, vegetables had a significantly higher aggregate concentration of residues than fruits, and this was expected, since vegetables (leafy ones in particular) are physiologically more prone to pesticide pollution than fruit crops [19,20]. The differences in residue concentrations can be partially explained by the differences in the persistence and dissipation rates of these residues, as well as weather conditions. Furthermore, the application rates between the different regions, according to the recommendations and farm size, could also potentially explain the differences in concentration across regions.

Similar studies have been conducted elsewhere. In Dhaka, Bangladesh, pesticide residues and OCPs, in particular, have been detected in 45% of vegetables and 40% of fruits examined, much lower than the detection rate observed in the present study [21]. In Changchun, Northeast China, the pesticides detection rate was also lower at 47–71% [20]. A 2011 study in Accra metropolis, Ghana, revealed that 51% of the fruits and vegetables analyzed gave results of traces of pesticide residues. In neighboring Qatar, the findings were similar to the present study in that at least one OCP residue was found in the fruits and vegetables investigated [22]. The variations in the pesticide detection rates in other developing countries may be partially explained by the differences in the type of compounds included in the investigation, as well as the fruits and vegetables included in the sample analysis, as some of these crops are geographic- and season-sensitive. In all cases, the list of crops and compounds of interest are massive enough to have meaningful comparisons across nations. 

Cypermethrin is the most commonly found pesticide residue in all the samples measured. It is also the most commonly used pyrethroid to control many pests [23]. Consequently, it is also the most frequently identified pyrethroid in vegetables and fruits in China [24]. Pyrethroids have low toxicity and persistence as compared to other insecticides, but cypermethrin remains highly lipophilic and, therefore, has easier access to the blood–brain barrier, which can directly cause damage in the central nervous system [25]. In fact, in a recent review appraising 27 studies on the effects of pesticides among agricultural workers suffering from symptoms of depression, 11 of such studies investigated pyrethroid pesticides, and, over-all, 78% of the studies included showed a clear link between pesticide exposure and depressive symptoms [26]. Furthermore, in a study done in Morocco on the effects of illegal dosing of cypermethrin by local farmers, vegetables exposed to higher than the allowed doses of cypermethrin were observed to have substantially lower antioxidant activity as compared to those exposed to legal doses [27]. The effects of cypermethrin toxicity can range from mild local symptoms like paresthesia following dermal contamination to neurological symptoms like seizures, fasciculation, tremors, coma and gastrointestinal symptoms like nausea, vomiting and gastrointestinal irritation and prolonged bradycardia [28,29].

In the present study, although pyraclostrobin was detected in only three samples, its concentrations were the highest among all residues measured. Pyraclostrobin is a heavily used fungicide in crops and was recently observed to cause deleterious effects in humans, including neurodegeneration and triglyceride build-up [30]. This compound belongs under the category of strobilurin fungicides, which are considered mitochondrial respiration inhibitors. Hence, it has the ability to disrupt ATP (adenosine triphosphate) synthesis, as observed in several organisms, especially aquatic animals, where it tends to bioaccumulate [31]. In a toxicological summary provided by the Minnesota Department of Health, pyraclostrobin toxicity may cause some degree of reproductive and developmental abnormalities in mammals, while the Centers for Disease Control and Prevention (CDC) considers this compound a mere skin and eye irritant [32]. The detection of pyraclostrobin in high concentrations in a few samples in the present study warrants close monitoring from authorities, since the detection was made in all regions except the western part, indicating the spread of its utilization kingdom-wide. Given that the Saudi diet considers fish as one of its main staple dishes [33], pyraclostrobin monitoring in fishes sold in local markets would be an interesting area of future investigation.

It is important to mention that, in the present study, some of the detected pesticide residues—such as cypermethrin, together with chlorpyrifos and fipronil—are restricted pesticides in KSA, as classified by the Saudi Food and Drug Administration (SFDA), and their use is only authorized to certified individuals [34]. This may partially explain why chlorpyrifos and fipronil, among other residues investigated, were not detected in the fruits and vegetables coming from the western region and, hence, have lower detection rates and aggregate residue concentration. In contrast, cypermethrin concentration and detection rates were highest in the western and northern regions (Table S1), which may mean several things: that permission to use this restricted pesticide is concentrated in these regions, that certain pests most reactive to cypermethrin are more prevalent in these regions, and/or more vegetable crops are grown in these areas since the concentration of cypermethrin was also significantly higher in vegetables than fruits investigated (Table S1), hence the higher detection rate. Furthermore, none of the banned pesticides, as classified by the SFDA (e.g., carbofuran, lindane, etc.…), were detected in all regions, indicating that local farmers, to some extent, were compliant in terms of good agricultural practices (GAP).

The authors acknowledge some limitations. The study was limited to the assessment of pesticide residues present in fruits and vegetables only, and consumption health risk was not included. As such, risk of exposure due to consumption cannot be ascertained. Worthy to note is that fruit and vegetable consumption in KSA overall is low, with one study indicating that only 2.6% of almost 11,000 Saudis aged 15 and above who participated met the daily guidelines for daily fruit and vegetable consumption [35]. Still, the omnipresence of pesticide residues is not limited to vegetables and fruits, and other diets, such as meat and dairy products, as well as drinking water, should also be assessed in future investigations. 

Nevertheless, the present study documents an extensive list of pesticide residues that were never before investigated in KSA agriculture products, filling the needed gap that will help policy makers fulfill their role in the International Code of Conduct on Pesticide Management and its obligations in the food safety of the Saudi community. An added strength of the study is the robustness of the methods used to determine pesticide residues, which were confirmed externally by collaborating laboratories outside KSA. Since exposure to pesticides can only be minimized at best, public health campaigns to reduce exposure and consumption by simple washing, trimming and selecting a variety of fruits and vegetables as suggested by the U.S. Environmental Protection Agency may be potent strategies to decrease health risks. Active and regular biomonitoring of crops should also be implemented, not only by SFDA to ensure food safety, but also by the academic community, such as the present study, to ensure accuracy of risk and level of contamination.

## 5. Conclusions

In conclusion, there is a high detection rate of pesticide residues in major regions in KSA, the majority of which were under the moderately hazardous type, and these were more common in vegetables than fruits. Although intake risk assessment was not carried out in the present study, consumption of vegetables and fruits purchased in KSA may still pose a risk to human health and, as such, caution should be exercised. Other potential sources of pesticide contamination, such as water, soil, meat and aquamarine products, should also be investigated to estimate the extent of human exposure in Saudi settings. Meanwhile, closer monitoring of local agricultural products, as well as agricultural practices, is needed to ultimately lessen the use of more hazardous pesticides, as well as public health campaigns to reduce exposure and consumption of these compounds.

## Figures and Tables

**Table 1:** Differences in pesticide residue distribution across regions according to Food Type and Season.

Pesticide Residues		Regions (N = 392)				*p*-Values
		Western	Central	Northern	Eastern	
N		41	146	131	74	
Overall	Absent	12 (29.3)	15 (10.3)	15 (11.5)	12 (16.2)	0.01
	Present	29 (70.7)	131 (89.7)	116 (88.5) ^A^	62 (83.8)	
Fruits	Absent	3 (27.3)	4 (6.6)	4 (9.1)	2 (6.1)	0.24
	Present	8 (72.7)	57 (93.4)	40 (90.9)	31 (93.9)	
Vegetables	Absent	9 (30.0)	11 (12.9)	11 (12.6)	10 (24.4)	0.06
	Present	21 (70.0)	74 (87.1)	76 (87.4)	31 (75.6)	

Note: Data presented as N (%); Superscript ^A^ indicates significance from western region; significance at *p* < 0.05.

**Table 2:** Regional concentration of pesticide residues.

Pesticide Residue (mg/kg)	Toxicity	Overall	Western	Central	Northern	Eastern	*p*-Value
∑Residues	--	0.15 ± 0.02	0.14 ± 0.09	0.10 ± 0.02	0.20 ± 0.05 ^A^	0.16 ± 0.07	0.001
2-phenylphenol	Class III	0.01 ± 0.0		0.01 ± 0.00	0.01	0.01 ± 0.00	0.47
Abamectin	Class Ib	0.02 ± 0.0		0.01 ± 0.01	0.02 ± 0.00		0.81
Acetamiprid	Class II	0.37 ± 0.16		0.33 ± 0.29	0.09 ± 0.04	0.76 ± 0.39	0.42
Azoxystrobin	Class U	0.02 ± 0.01		0.01 ± 0.00	0.03 ± 0.02	<0.01 ± 0.00	0.22
Bifenazate	Class U	0.07 ± 0.05	0.02	0.09 ± 0.07	0.02		0.93
Bifenthrin	Class II	0.15 ± 0.01			0.17	0.14	0.32
Boscalid	Class U	0.06 ± 0.03	0.01	0.10 ± 0.07	0.03 ± 0.02	0.08 ± 0.08	0.43
Bupirimate	Class III	0.02 ± 0.01	0.02	0.05	<0.01	<0.01	0.39
Chlorantraniliprole	Class U	0.04 ± 0.02		0.04	0.04 ± 0.03		1
Chlorpyrifos	Class II	0.17 ± 0.11		0.23 ± 0.23	0.15 ± 0.07	0.04 ± 0.04	0.2
Clothianidin	Class II	0.09 ± 0.08	0.01	0.18			0.32
Cypermethrin	Class II	0.19 ± 0.10	0.28 ± 0.27	0.01 ± 0.01	0.27 ± 0.17	0.03 ± 0.01	0.72
Deltamethrin	Class II	0.06 ± 0.02		0.04 ± 0.02	0.10 ± 0.05	0.02 ± 0.01	0.3
Difenoconazole	Class II	0.04 ± 0.03		0.07 ± 0.07	0.01 ± 0.00	0.03 ± 0.02	0.96
Dinotefuran	Class III	0.02 ± 0.0		0.02 ± 0.00	0.03		0.22
Emamectin	Class II	0.06 ± 0.02		0.1	0.05 ± 0.03		0.32
Fenbuconazole	Class III	0.09 ± 0.05		0.01	0.12 ± 0.05		0.22
Fipronil	Class II	0.29 ± 0.27		0.43 ± 0.41		0.02	0.22
Fludioxonil	Class U	0.33 ± 0.12		0.20 ± 0.13	0.30 ± 0.15	0.76 ± 0.54	0.43
Fluopyram	Class III	0.17 ± 0.06	0.08		0.18 ± 0.17	0.19 ± 0.07	0.74
Imazalil	Class II	0.14 ± 0.04	0.04 ± 0.02	0.07 ± 0.03	0.26 ± 0.10	0.10 ± 0.02	0.21
Imidacloprid	Class II	0.07 ± 0.03	0.02 ± 0.01	0.09 ± 0.05	0.08 ± 0.06	0.04 ± 0.02	0.9
Indoxacarb	Class II	0.53 ± 0.22	1.37 ± 1.36	0.28 ± 0.28	0.53 ± 0.27	0.01 ± 0.00	0.47
Lambda-Cyhalothrin	Class II	0.03 ± 0.01		0.04 ± 0.02	0.04 ± 0.03	0.01 ± 0.01	0.61
Metalaxyl	Class II	0.02 ± 0.01	0.01	0.01 ± 0.01	0.03 ± 0.02	<0.01	0.76
Pendimethalin	Class II	0.01 ± 0.01		<0.01	0.03		0.32
Pirimicarb	Class II	0.02 ± 0.0	0.02	0.02			0.32
Pyraclostrobin	Class II	1.76 ± 1.34		0.86	4.4	0.02	0.37
Pyrimethanil	Class III	0.47 ± 0.22	0.01 ± 0.01	0.27 ± 0.26	0.49 ± 0.27	1.04 ± 0.99	0.03
Tebuconazole	Class II	0.03 ± 0.01		0.05 ± 0.02	0.01 ± 0.00	0.02 ± 0.01	0.69
Tetraconazole	Class II	0.01 ± 0.0	0.01		0.01		0.32
Thiabendazole	Class III	0.03 ± 0.0		0.02 ± 0.01	0.04 ± 0.00	0.01 ± 0.00	0.09
Thiamethoxam	Class II	0.03 ± 0.01	0.01 ± 0.00	0.04 ± 0.01	0.02 ± 0.01	0.03 ± 0.01	0.19

Note: Data presented as mean ± SE; *p*-values are obtained from Kruskal–Wallis test; a *p*-value < 0.05 is considered significant. Superscript ^A^ indicates significance compared to the western region.

**Table 3:** Concentration of pesticide residues according to food type.

Pesticide Residue (mg/kg)	Toxicity	Fruits	Vegetables	*p*-Value
∑Residues		0.13 ± 0.04	0.16 ± 0.03	0.02
2-phenylphenol	Class III	0.01 ± 0.00	0.01 ± 0.00	1
Abamectin	Class Ib	0.01	0.02 ± 0.00	0.55
Acetamiprid	Class II	0.07 ± 0.05	0.58 ± 0.25	0.08
Azoxystrobin	Class U	0.05 ± 0.05	0.01 ± 0.00	0.82
Bifenazate	Class U	0.11 ± 0.10	0.03 ± 0.00	0.51
Bifenthrin	Class II	0.17	0.14	0.32
Boscalid	Class U	0.06 ± 0.03	0.06 ± 0.06	0.46
Carbendazim	Class U	0.02	0.01	0.32
Chlorantraniliprole	Class U	0.04	0.04 ± 0.03	1
Chlorpyrifos	Class II	0.05 ± 0.03	0.32 ± 0.26	0.16
Cypermethrin	Class II	0.01 ± 0.00	0.22 ± 0.11	0.05
Deltamethrin	Class II	0.03 ± 0.01	0.08 ± 0.04	0.46
Difenoconazole	Class II	0.02 ± 0.01	0.05 ± 0.05	0.65
Emamectin	Class II	0.03	0.08 ± 0.03	0.32
Fenbuconazole	Class III	0.12 ± 0.05	0.01	0.22
Fludioxonil	Class U	0.36 ± 0.15	0.20 ± 0.10	0.75
Fluopyram	Class III	0.31 ± 0.04	0.07 ± 0.03	0.08
Imazalil	Class II	0.15 ± 0.05	0.12 ± 0.07	0.44
Imidacloprid	Class II	0.24	0.05 ± 0.02	0.16
Indoxacarb	Class II	0.58	0.52 ± 0.23	0.43
Lambda-Cyhalothrin	Class II	0.02 ± 0.01	0.04 ± 0.02	1
Malathion	Class III	<0.01	0.21 ± 0.20	0.22
Myclobutanil	Class II	0.02 ± 0.02	0.05	0.22
Pendimethalin	Class II	0.03	<0.01	0.32
Pyraclostrobin	Class II	0.02	2.63 ± 1.77	0.22
Pyrimethanil	Class III	0.51 ± 0.29	0.37 ± 0.29	0.89
Tebuconazole	Class II	0.02 ± 0.01	0.03 ± 0.01	1
Thiabendazole	Class III	0.02 ± 0.01	0.03 ± 0.01	0.71
Thiamethoxam	Class II	0.04 ± 0.03	0.03 ± 0.01	0.34
Thiophanate-methyl	Class U	0.01	0.02	0.32
Trifloxystrobin	Class U	<0.01	0.01 ± 0.00	0.48

Note: Data presented as mean ± SE; *p*-values are obtained from Mann–Whitney U-test; *p*-value < 0.05 considered significant.

## Data Availability

The data presented in this study are available on request from the corresponding author. The data are not publicly available due to privacy protection.

## Supplementary Materials

The following supporting information can be downloaded at: https://www.mdpi.com/article/10.3390/toxics11090798/s1, Table S1: Toxicity of pesticide residues assessed according to WHO and EPA classification hazard; Table S2: Prevalence of pesticide residue according to region and food type; Table S3: Concentration of pesticide residues limited to only one category of region; Table S4: Concentration of pesticide residues that were limited to only one category of food type.
