# Supplementary material for: Regional Variations in Pesticide Residue Detection Rates and Concentrations in Saudi Arabian Crops
Source: Toxics. 2023 Sep 21;11(9):798. doi: 10.3390/toxics11090798 (PMC10537341; doi:10.3390/toxics11090798)
Supplement: Supplementary file 1 [file toxics-11-00798-s001.zip › toxics-2599109-supplementary.pdf]

Table S1. Toxicity of Pesticide Residues Assessed according to WHO and EPA hazard classification

**Pesticide residue**	**Toxicity**	
2-phenylphenol	Class III	Slightly hazardous
Abamectin	Class Ib	Highly hazardous
Acetamiprid	Class II	Moderately hazardous
Azoxystrobin	Class U	Unlikely to present acute hazard
Bifenazate	Class U	Unlikely to present acute hazard
Bifenthrin	Class II	Moderately hazardous
Boscalid	Class U	Unlikely to present acute hazard
Bupirimate	Class III	Slightly hazardous
Carbendazim	Class U	Unlikely to present acute hazard
Chlorantraniliprole	Class U	Unlikely to present acute hazard
Chlorfenapyr	Class II	Moderately hazardous
Chlorpyrifos	Class II	Moderately hazardous
Clothianidin	Class II	Moderately hazardous
Cypermethrin	Class II	Moderately hazardous
Cyprodinil	Class III EPA	Slightly hazardous
Deltamethrin	Class II	Moderately hazardous
Diazinon	Class II	Moderately hazardous
Difenoconazole	Class II	Moderately hazardous
Dinotefuran	Class III	Slightly hazardous
Emamectin	Class II	Moderately hazardous
Ethoprophos	Class Ia	Extremely hazardous
Etofenprox	Class U	Unlikely to present acute hazard
Fenamiphos	Class Ib	Highly hazardous
Fenbuconazole	Class III	Slightly hazardous
Fenhexamid	Class U	Unlikely to present acute hazard
fenpropimorph	Class III	Slightly hazardous
Fenpyroximate	Class II	Moderately hazardous
Fipronil	Class II	Moderately hazardous
Fludioxonil	Class U	Unlikely to present acute hazard
Fluopyram	Class III	Slightly hazardous
Flutriafol	Class II	Moderately hazardous
Imazalil	Class II	Moderately hazardous
Imidacloprid	Class II	Moderately hazardous
Indoxacarb	Class II	Moderately hazardous
Lambda-Cyhalothrin	Class II	Moderately hazardous
Malathion	Class III	Slightly hazardous
Metalaxyl	Class II	Moderately hazardous

Methomyl	Class Ib	Highly hazardous
Metribuzin	Class II	Moderately hazardous
Myclobutanil	Class II EPA	Moderately hazardous.
Penconazole	Class III	Slightly hazardous
Pendimethalin	Class II	Moderately hazardous
Phosmet	Class II	Moderately hazardous
Pirimicarb	Class II	Moderately hazardous
Pirimicarb desmethyl	Class II	Moderately hazardous
Propamocarb	Class U	Unlikely to present acute hazard
Propargite	Class III	Slightly hazardous
Propiconazole	Class II	Moderately hazardous
Pyraclostrobin	Class II	Moderately hazardous
Pyridaben	Class II	Moderately hazardous
Pyrimethanil	Class III	Slightly hazardous
Pyriproxyfen	Class U	Unlikely to present acute hazard
Spirodiclofen	Class III	Slightly hazardous
Tebuconazole	Class II	Moderately hazardous
Tetraconazole	Class II	Moderately hazardous
Tetramethrin	Class U	Unlikely to present acute hazard
Thiabendazole	Class III	Slightly hazardous
Thiamethoxam	Class II	Moderately hazardous
Thiophanate-methyl	Class U	Unlikely to present acute hazard
Triadimenol	Class II	Moderately hazardous
Trifloxystrobin	Class U	Unlikely to present acute hazard

Note: Classification is based on oral and Dermal LD_{50} for the rat (mg/kg body weight)

Table S2. Prevalence of Pesticide Residues according to region and food type

Pesticide Residues	All	Western	Central	Northern	Eastern	*p*-Value	Fruits	Vegetables	*p*-Value
	**392**	**41**	**146**	**131**	**74**		**149**	**243**	
**Absent**	54 (13.8)	12 (29.3)	15 (10.3)	15 (11.5)	12 (16.2)	0.01	13 (8.7)	41 (16.9)	0.02
2-phenylphenol	5 (1.3)	0 (0.0)	2 (1.4)	1 (0.8)	2 (2.7)	0.57	3 (2.0)	2 (0.8)	0.31
Abamectin	6 (1.5)	0	2 (1.4)	4 (3.1)	0 (0.0)	0.28	1 (0.7)	5 (2.1)	0.28
Acetamiprid	17 (4.3)	0	6 (4.1)	6 (4.6)	5 (6.8)	0.4	7 (4.7)	10 (4.1)	0.78
Azoxystrobin	10 (2.6)	0	2 (1.4)	6 (4.6)	2 (2.7)	0.25	3 (2.0)	7 (2.9)	0.6
Bifenazate	6 (1.5)	1 (2.4)	4 (2.7)	1 (0.8)	0	0.35	3 (2.0)	3 (1.2)	0.54
Bifenthrin	2 (0.5)	0	0 (0.0)	1 (0.8)	1 (1.4)	0.54	1 (0.7)	1 (0.4)	0.73
Boscalid	9 (2.3)	1 (2.4)	3 (2.1)	3 (2.3)	2 (2.7)	0.99	5 (3.4)	4 (1.6)	0.27
Bupirimate	4 (1.0)	1 (2.4)	1 (0.7)	1 (0.8)	1 (1.4)	0.77	4 (2.7)	0	0.01
Carbendazim	2 (0.5)	0	2 (1.4)	0	0	0.34	1 (0.7)	1 (0.4)	0.73
Chlorantraniliprole	3 (0.8)	0	1 (0.7)	2 (1.5)	0	0.59	1 (0.7)	2 (0.8)	0.87
Chlorfenapyr	1 (0.3)	0	0 (0.0)	1 (0.8)	0	0.57	0	1 (0.4)	0.43
Chlorpyrifos	12 (3.1)	0	6 (4.1)	3 (2.3)	3 (4.1)	0.5	7 (4.7)	5 (2.1)	0.14
Clothianidin	2 (0.5)	1 (2.4)	1 (0.7)	0	0	0.25	0	2 (0.8)	0.27
Cypermethrin	32 (8.2)	9 (22.0)	6 (4.1)	12 (9.2)	5 (6.8)	0.002	5 (3.4)	27 (11.1)	0.006
Cyprodinil	1 (0.3)	0	1 (0.7)	0	0	0.64	1 (0.7)	0	0.2
Deltamethrin	9 (2.3)	0	4 (2.7)	3 (2.3)	2 (2.7)	0.76	4 (2.7)	5 (2.1)	0.69
Diazinon	1 (0.3)	0	0	1 (0.8)	0	0.57	1 (0.7)	0	0.2
Difenoconazole	10 (2.6)	0	5 (3.4)	3 (2.3)	2 (2.7)	0.67	3 (2.0)	7 (2.9)	0.6
Dinotefuran	3 (0.8)	0	2 (1.4)	1 (0.8)	0	0.66	0	3 (1.2)	0.17
Emamectin	4 (1.0)	0	1 (0.7)	3 (2.3)	0	0.33	1 (0.7)	3 (1.2)	0.59
Ethoprophos	1 (0.3)	0	0	1 (0.8)	0	0.57	1 (0.7)	0	0.2
Etofenprox	1 (0.3)	0	1 (0.7)	0	0	0.64	0	1 (0.4)	0.43
Fenamiphos	1 (0.3)	0	0	1 (0.8)	0	0.57	1 (0.7)	0	0.2
Fenbuconazole	3 (0.8)	0	1 (0.7)	2 (1.5)	0 (0.0)	0.59	2 (1.3)	1 (0.4)	0.3

Fenhexamid	1 (0.3)	0	1 (0.7)	0	0	0.64	0	1 (0.4)	0.43
Fenpropimorph	1 (0.3)	0	0	0	1 (1.4)	0.23	1 (0.7)	0	0.2
Fenpyroximate	1 (0.3)	0	0	0	1 (1.4)	0.23	1 (0.7)	0	0.2
Fipronil	3 (0.8)	0	2 (1.4)	0	1 (1.4)	0.5	0	3 (1.2)	0.17
Fludioxonil	17 (4.3)	0	8 (5.5)	6 (4.6)	3 (4.1)	0.5	14 (9.4)	3 (1.2)	<0.001
Fluopyram	5 (1.3)	1 (2.4)	0 (0.0)	2 (1.5)	2 (2.7)	0.31	2 (1.3)	3 (1.2)	0.93
Flutriafol	1 (0.3)	0	1 (0.7)	0	0	0.64	1 (0.7)	0	0.2
Imazalil	18 (4.6)	2 (4.9)	6 (4.1)	6 (4.6)	4 (5.4)	0.98	11 (7.4)	7 (2.9)	0.04
Imidacloprid	15 (3.8)	2 (4.9)	5 (3.4)	5 (3.8)	3 (4.1)	0.98	1 (0.7)	14 (5.8)	0.01
Indoxacarb	20 (5.1)	2 (4.9)	3 (2.1)	13 (9.9)	2 (2.7)	0.02	1 (0.7)	19 (7.8)	0.001
Lambda-Cyhalothrin	8 (2.0)	0	4 (2.7)	2 (1.5)	2 (2.7)	0.67	4 (2.7)	4 (1.6)	0.48
Malathion	3 (0.8)	0	3 (2.1)	0	0	0.16	1 (0.7)	2 (0.8)	0.87
Metalaxyl	9 (2.3)	1 (2.4)	5 (3.4)	2 (1.5)	1 (1.4)	0.69	0 (0)	9 (3.7)	0.02
Methomyl	1 (0.3)	0	0	1 (0.8)	0	0.57	0	1 (0.4)	0.43
Metribuzin	1 (0.3)	0	0	1 (0.8)	0	0.57	0	1 (0.4)	0.43
Myclobutanil	3 (0.8)	0	3 (2.1)	0	0	0.16	2 (1.3)	1 (0.4)	0.3
Penconazole	1 (0.3)	0	1 (0.7)	0	0	0.64	0	1 (0.4)	0.43
Pendimethalin	2 (0.5)	0	1 (0.7)	1 (0.8)	0	0.84	1 (0.7)	1 (0.4)	0.73
Phosmet	1 (0.3)	0	0	0	1 (1.4)	0.23	1 (0.7)	0	0.2
Pirimicarb	2 (0.5)	1 (2.4)	1 (0.7)	0	0	0.25	2 (1.3)	0	0.07
Pirimicarb desmethyl	1 (0.3)	0	1 (0.7)	0	0	0.64	1 (0.7)	0	0.2
Propamocarb	1 (0.3)	0	0	0	1 (1.4)	0.23	0	1 (0.4)	0.43
Propargite	1 (0.3)	0	1 (0.7)	0	0	0.64	0	1 (0.4)	0.43
Propiconazole	1 (0.3)	0	1 (0.7)	0	0	0.64	0	1 (0.4)	0.43
Pyraclostrobin	3 (0.8)	0	1 (0.7)	1 (0.8)	1 (1.4)	0.88	1 (0.7)	2 (0.8)	0.87
Pyridaben	1 (0.3)	0	0	1 (0.8)	0	0.57	1 (0.7)	0	0.2
Pyrimethanil	22 (5.6)	2 (4.9)	8 (5.5)	8 (6.1)	4 (5.4)	0.99	15 (10.1)	7 (2.9)	0.002
Pyriproxyfen	1 (0.3)	0	0	1 (0.8)	0	0.57	0	1 (0.4)	0.43

Spirodiclofen	1 (0.3)	0	1 (0.7)	0	0	0.64	1 (0.7)	0	0.2
Tebuconazole	9 (2.3)	0	4 (2.7)	3 (2.3)	2 (2.7)	0.76	2 (1.3)	7 (2.9)	0.32
Tetraconazole	2 (0.5)	1 (2.4)	0 (0.0)	1 (0.8)	0	0.23	2 (1.3)	0	0.07
Tetramethrin	1 (0.3)	0	1 (0.7)	0	0	0.64	0	1 (0.4)	0.43
Thiabendazole	12 (3.1)	0	5 (3.4)	3 (2.3)	4 (5.4)	0.39	9 (6.0)	3 (1.2)	<0.001
Thiamethoxam	18 (4.6)	4 (9.8)	7 (4.8)	3 (2.3)	4 (5.4)	0.24	3 (2.0)	15 (6.2)	0.06
Thiophanate-methyl	2 (0.5)	0	2 (1.4)	0	0	0.34	1 (0.7)	1 (0.4)	0.73
Triadimenol	1 (0.3)	0	1 (0.7)	0	0	0.64	1 (0.7)	0	0.2
Trifloxystrobin	3 (0.8)	0	3 (2.1)	0	0	0.16	1 (0.7)	2 (0.8)	0.87

Note: Data presented as N (%).

Table S3: Concentration of Pesticide Residues limited to only one category of Region

Pesticide Residues (mg/kg)	Overall	Western	Central	Northern	Eastern
Carbendazim	0.02 ± 0.00		0.02 ± 0.00		
Chlorfenapyr	0.01			0.01	
Cyprodinil	0.03		0.03		
Diazinon	0.01			0.01	
Ethoprophos	0.01			0.01	
Etofenprox	0.12		0.12		
Fenamiphos	0.19			0.19	
Fenhexamid	0.01		0.01		
Fenpyroximate	0.03				0.03
Flutriafol	0.01		0.01		
Malathion	0.14 ± 0.13		0.14 ± 0.13		
Methomyl	0.01			0.01	
Myclobutanil	0.03 ± 0.01		0.03 ± 0.01		
Phosmet	0.01				0.01
Pirimicarb desmethyl	0.27		0.27		
Propamocarb	0.05				0.05
Propargite	0.01		0.01		
Propiconaz	0.02		0.02		
Pyridaben	0.03			0.03	
Pyriproxyfen	0.01			0.01	
Spirodiclofen	0.15		0.15		
Thiophanate-methyl	0.02 ± 0.01		0.02 ± 0.01		
Triadimenol	0.04		0.04		
Trifloxystrobin	0.00 ± 0.00		0.00 ± 0.00		

Note: Data presented as Mean ± SE when two or more samples are available.

Table S4. Concentration of Pesticide Residues that were limited to only one category of food type.

**Pesticide Residues (mg/kg)**	**Fruits**	**Vegetables**
Bupirimate	0.02 ± 0.01	
Chlorfenapyr		0.01
Clothianidin		0.09 ± 0.08
Cyprodinil	0.03 ±	
Diazinon	0.01	
Dinotefuran		0.02 ± 0.00
Ethoprophos	0.01	
Etofenprox		0.12
Fenamiphos	0.19	
Fenhexamid		0.01
Fenpropimorph	<0.01	
Fenpyroximate	0.03	
Fipronil		0.29 ± 0.27
Flutriafol	0.01	
Metalaxyl		0.02 ± 0.01
Methomyl		0.01
Metribuzin		<0.01
Penconazole		<0.01
Phosmet	0.01	
Pirimicarb	0.02 ± 0.00	
Pirimicarb desmethyl	0.27	
Propamocarb		0.05
Propargite		0.01
Propiconazole		0.02
Pyridaben	0.03	
Pyriproxyfen		0.01
Spirodiclofen	0.15	
Tetraconazole	0.01 ± 0.0	
Tetramethrin		<0.01
Triadimenol	0.04	

Note: Data presented as Mean ± SE when two or more samples are available.
